# Extracellular cold-inducible RNA-binding protein mediated neuroinflammation and neuronal apoptosis after traumatic brain injury

**DOI:** 10.1093/burnst/tkae004

**Published:** 2024-05-29

**Authors:** Yu-xiao Liu, Ming Zhao, Yang Yu, Jing-peng Liu, Wen-jia Liu, Ren-qi Yao, Jing Wang, Rong-li Yang, Yao Wu, Ning Dong, Yang Cao, Shou-chun Li, Qin-hong Zhang, Run-min Yan, Yong-ming Yao

**Affiliations:** Department of Neurosurgery, First Medical Center of the Chinese PLA General Hospital, Beijing 100853, People’s Republic of China; Department of Neurosurgery, First Medical Center of the Chinese PLA General Hospital, Beijing 100853, People’s Republic of China; Department of Traditional Chinese Medical Science, Sixth Medical Center of the Chinese PLA General Hospital, Beijing 100037, People’s Republic of China; Department of Traditional Chinese Medical Science, Sixth Medical Center of the Chinese PLA General Hospital, Beijing 100037, People’s Republic of China; State Key Laboratory of Proteomics, Beijing Proteome Research Center, National Center for Protein Sciences, Beijing Institute of Lifeomics, Beijing 100071, People’s Republic of China; Translational Medicine Research Center, Medical Innovation Research Division and Fourth Medical Center of the Chinese PLA General Hospital, Beijing 100853, People’s Republic of China; Department of Obstetrics and Gynecology, Seventh Medical Center of the Chinese PLA General Hospital, Beijing 100700, People’s Republic of China; Intensive Care Unit, Dalian Municipal Central Hospital Affiliated Dalian University of Technology, Dalian 116033, People’s Republic of China; Translational Medicine Research Center, Medical Innovation Research Division and Fourth Medical Center of the Chinese PLA General Hospital, Beijing 100853, People’s Republic of China; Translational Medicine Research Center, Medical Innovation Research Division and Fourth Medical Center of the Chinese PLA General Hospital, Beijing 100853, People’s Republic of China; Department of Neurosurgery, First Medical Center of the Chinese PLA General Hospital, Beijing 100853, People’s Republic of China; Department of Neurosurgery, First Medical Center of the Chinese PLA General Hospital, Beijing 100853, People’s Republic of China; Translational Medicine Research Center, Medical Innovation Research Division and Fourth Medical Center of the Chinese PLA General Hospital, Beijing 100853, People’s Republic of China; Department of Neurosurgery, First Medical Center of the Chinese PLA General Hospital, Beijing 100853, People’s Republic of China; Translational Medicine Research Center, Medical Innovation Research Division and Fourth Medical Center of the Chinese PLA General Hospital, Beijing 100853, People’s Republic of China

**Keywords:** Traumatic brain injury, Neuroinflammation, Apoptosis, Cold-inducible RNA-binding protein, Endoplasmic reticulum stress, Histone H3 acetylation, Fluid percussion injury, Ischaemia

## Abstract

**Background:**

Extracellular cold-inducible RNA-binding protein (eCIRP) plays a vital role in the inflammatory response during cerebral ischaemia. However, the potential role and regulatory mechanism of eCIRP in traumatic brain injury (TBI) remain unclear. Here, we explored the effect of eCIRP on the development of TBI using a neural-specific CIRP knockout (KO) mouse model to determine the contribution of eCIRP to TBI-induced neuronal injury and to discover novel therapeutic targets for TBI.

**Methods:**

TBI animal models were generated in mice using the fluid percussion injury method. Microglia or neuron lines were subjected to different drug interventions. Histological and functional changes were observed by immunofluorescence and neurobehavioural testing. Apoptosis was examined by a TdT-mediated dUTP nick end labelling assay *in vivo* or by an annexin-V assay *in vitro*. Ultrastructural alterations in the cells were examined via electron microscopy. Tissue acetylation alterations were identified by non-labelled quantitative acetylation via proteomics. Protein or mRNA expression in cells and tissues was determined by western blot analysis or real-time quantitative polymerase chain reaction. The levels of inflammatory cytokines and mediators in the serum and supernatants were measured via enzyme-linked immunoassay.

**Results:**

There were closely positive correlations between eCIRP and inflammatory mediators, and between eCIRP and TBI markers in human and mouse serum. Neural-specific eCIRP KO decreased hemispheric volume loss and neuronal apoptosis and alleviated glial cell activation and neurological function damage after TBI. In contrast, eCIRP treatment resulted in endoplasmic reticulum disruption and ER stress (ERS)-related death of neurons and enhanced inflammatory mediators by glial cells. Mechanistically, we noted that eCIRP-induced neural apoptosis was associated with the activation of the protein kinase RNA-like ER kinase-activating transcription factor 4 (ATF4)-C/EBP homologous protein signalling pathway, and that eCIRP-induced microglial inflammation was associated with histone H3 acetylation and the α7 nicotinic acetylcholine receptor.

**Conclusions:**

These results suggest that TBI obviously enhances the secretion of eCIRP, thereby resulting in neural damage and inflammation in TBI. eCIRP may be a biomarker of TBI that can mediate the apoptosis of neuronal cells through the ERS apoptotic pathway and regulate the inflammatory response of microglia via histone modification.

## Graphical Abstract

**Graphical Abstract:**
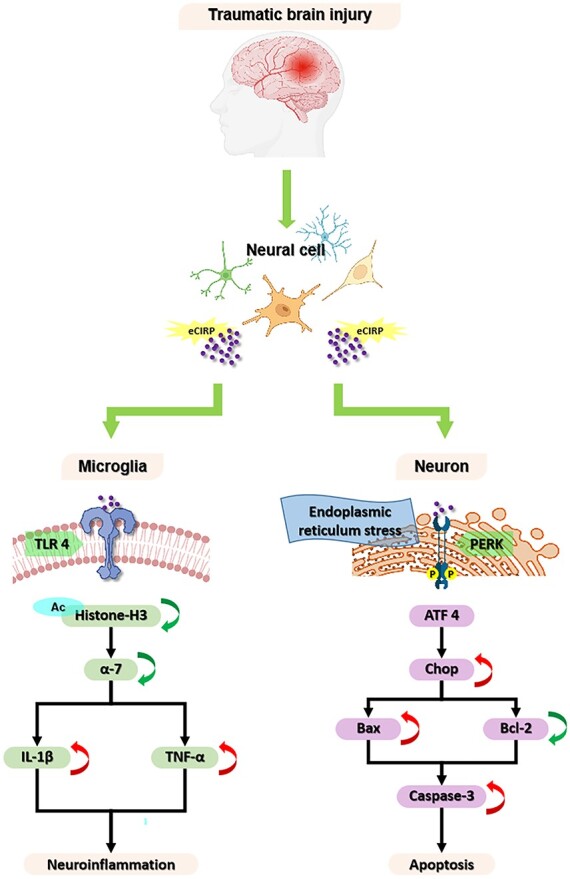

Highlights

- eCIRP is induced by brain injury and might be used as a biomarker of TBI.
- eCIRP may be a vital contributor to neuron apoptosis and the inflammatory response of microglia during TBI.
- eCIRP induces neural apoptosis via the PERK–ATF4–CHOP signaling pathway.
- eCIRP regulates microglial polarization via histone modification.
- CIRP deficiency alleviates the behavioural deficits induced by TBI.

## Background

Approximately 69 million people suffer from traumatic brain injury (TBI) annually worldwide [1,2]. TBI can progress through a process that includes primary injury and secondary craniocerebral injury. Secondary brain injury is a crucial factor for a worse prognosis in patients with TBI because it disrupts brain homeostasis and triggers a chronic neurodegenerative cascade [3,4].

Neuronal apoptosis and microglial polarization, the most common and vital cellular events following TBI, are the leading contributors to TBI-induced secondary injury [5,6]. Uncontrolled cell apoptosis appears to be an important cause of neurological disability in TBI [7]. Moreover, a persistent inflammatory response promotes neural cell death and exacerbates nerve function damage and posttraumatic disorders [8,9]. Notably, as the major resident immune cells of the brain, microglia are the main contributors to the pathogenesis of neuroinflammation. The M1-like phenotype of microglia is involved mainly in uncontrolled neuroinflammation. In contrast, the M2-like phenotype is associated with the alleviation of inflammation. Many studies have shown that inhibition of microglial activation markedly reduces neuroinflammation and thus alleviates long-term cognitive impairment following TBI [10,11]. Therefore, inhibiting neural apoptosis and modulating microglial M1/M2 polarization may be helpful for improving the functional outcomes of patients with TBI.

Cold-inducible RNA-binding protein (CIRP) was first discovered as an RNA chaperone that regulates the cell cycle in hibernating animals [12]. There are two different forms of CIRP: intracellular CIRP and eCIRP. Currently, increasing evidence has indicated that eCIRP is critically involved in the development of inflammatory diseases [13,14]. It is likely that eCIRP is associated with alcohol-, haemorrhage- and cerebral ischaemia-induced brain inflammation [15–18]. However, the role of CIRP in brain injury is still controversial. Wang *et al*. reported that CIRP inhibited apoptosis and exerted a neuroprotective effect during mild hypothermia in patients with TBI [19]. In contrast, Liu *et al*. demonstrated that CIRP deficiency relieves neuronal damage by inhibiting microglial activation during deep hypothermic circulatory arrest (DHCA) [20]. However, the potential role and mechanism of action of eCIRP in TBI have not been fully elucidated. We therefore aimed to explore the effects of eCIRP on neuronal apoptosis and microglial polarization during TBI and to define the underlying mechanisms of these phenomena.

## Methods

### Mice

Wild-type (WT) C57BL/6 J mice (male, weighing ~25 g) were obtained from the Institute of Laboratory Animal Science, Peking Union Medical College, Beijing, China. C57BL/6 neural-specific CIRP knockout (KO) mice (flox/flox, Nes-Cre) were purchased and were identified by Cyagen Biosciences Co., Guangzhou, China. Briefly, the Cas9 protein, two guide RNA (gRNAs) gRNAs (gRNA-1: TCCCACAGGCTAGGCGA GATGGG, gRNA-2: TTACTCGTTGTGTGTAGCTGAGG) and a donor vector containing two loxP sequences flanking exons 2–7 of mouse CIRP were coinjected into fertilized eggs. The embryos were subsequently transferred to recipient female mice to obtain F0 mice. The genotype of the conditional knockout mice was confirmed by PCR using two pairs of primers (F1: 5′-TTTTGGATTCTGTTCCTTTGCCTC-3′, R1: 5′-CTTC AAGTGGGGTTTCTTTCACAC-3′; F2: 5′-CACTGTCCCGCTTCTGTCCT-3′, R2: 5′-CTACAAAAGTGGGAACAAGGTGC-3′) and sequencing.

In these neural-specific CIRP KO mice, the CIRP gene was knocked out in central nervous system neurons and glial cell precursors, and CIRP was normally expressed in other tissues and cells.

Mice were housed in a room at a constant temperature on a 12-h light-to-dark cycle. All animal experiments were approved and carried out according to the guidelines of the ethical committee of the General Hospital of the Chinese PLA, Beijing, China (No. SYXK2019–0021).

### Patients

Venous blood was obtained from patients with a confirmed diagnosis of TBI 24 h after being in the hospital and from healthy donors with informed consent (Table 1). The details of the patients and the inclusion criteria are shown in Table 1. Briefly, 8 patients with a diagnosis of traumatic brain injury (TBI) in the Department of Neurosurgery, Chinese PLA General Hospital and 10 donors were enrolled in this retrospective study. The inclusion criteria for patients were as follows: (1) aged between 20 and 70 years, (2) diagnosed with moderate to severe TBI (Glasgow Coma Scale < 12), and (3) had a duration from injury to admission of <24 h. The exclusion criteria were as follows: (1) died within 24 h of admission, (2) had multiple traumatic conditions, including TBI, (3) had chronic intracranial haematoma, and (4) had a history of immune diseases. Blood samples were collected from patients and donors. Peripheral blood (2–5 ml) was collected in the morning, transferred to a blood procoagulation tube and gently inverted to mix fully. Blood samples were incubated at room temperature for 30 to 60 min and centrifuged at 13,000 rpm for 10 min at 4°C. The supernatants were stored at −70°C. This study was authorized by the ethics committee of Chinese PLA General Hospital, Beijing, China (No. S2021–539-01).

**Table 1:** Characteristics of TBI patients and volunteers

	**TBI patients**	**Healthy donors**
Gender		
Male Female	5 2	7 3
Age, years	30–66	26–64
Cause of TBI		
Traffic accident Fall	3 4	
Glasgow Coma Scale		
15 9–12 3–8	4 3	10
Subarachnoid haemorrhage	6	
Intracranial haemorrhage	7	

*TBI* traumatic brain injury

### Materials

GSK2656157 (inhibitor of protein kinase RNA-like ER kinase), TAK-242 and MC1742 were purchased from MedChemExpress Co., Shanghai, China. TRIzol reagent, iBlot, secondary antibodies conjugated to Cyanine (Cy)3 or Cy5, and SuperScript III reverse transcriptase were used. The secondary antibodies, chemiluminescence agent, and antibodies against CD86, β-actin and histone H3 (acetyl lys 9) were obtained from Santa Cruz Biotechnology, Santa Cruz, CA, USA. Antibodies against CIRP, glucose-regulated protein 78 (GRP78), transcription factor 4 (ATF4), CD206 and glial fibrillary acidic protein (GFAP). Ionized calcium binding adapter molecule-1 (Iba-1) and neuronal nuclei (NeuN) were purchased from Abcam, Cambridge, CA, USA. Antibodies against C/EBP homologous protein (CHOP), cleaved-caspase-3, caspase-3, B cell lymphoma gene-2 (Bcl-2), protein kinase RNA -like ER kinase (PERK), Bcl-2 associated X protein (Bax), cleaved protein kinase RNA-like ER kinase (p-PERK) and the acetyl-lysine motif kit were obtained from Cell Signaling Technology, Danvers, MA, USA. An Qproteome Mammalian Protein Prer Kit was obtained from QIAGEN GmbH (Germany). TdT-mediated dUTP nick end labelling (TUNEL) kits were purchased from Promega, Wisconsin, CA, USA. The primers and Small interfering RNA (si) CIRP or negative control (NC) of siCIRP were obtained from GenePharm Co., Suzhou, China. The BV2 and neuro-2a cell lines were purchased from Procell Life Science and Technology Co., Ltd, Wuhan, China. Triton X-100 was obtained from Sigma, St. Louis, MO, USA. Enzyme-linked immunosorbent (ELISA) kits were obtained from Excell Inc., Shanghai, China.

### The protocol of the experiments

In this study, animal experiments were carried out in mice with TBI. Cell experiments were performed using BV2 cells and neuro-2a cells. Details of the experiments are shown in supplementary Figure 1a, b, see online supplementary material.

#### In vivo

The TBI models were generated using the fluid percussion injury (FPI) method using WT and KO mice. The animals were then randomly divided into four groups: the WT sham group, the KO sham group, the WT TBI group and the KO TBI group. Details of the animal groups are shown in supplementary Figure 2a, see online supplementary material. Blood samples were collected from the WT sham and WT TBI groups on day post TBI (dpi) 1. Brain tissues were collected from different groups at 4 h and at 1, 7 and 28 dpi. Expression of CIRP, apoptosis-related proteins, inflammatory cytokines and glial cells markers were examined via western blotting (WB), real-time quantitative polymerase chain reaction (q-PCR) and immunofluorescence staining at various time-points after TBI. Apoptosis and brain damage were observed by haematoxylin and eosin (HE) staining and TUNEL assays at 4 h and at 1, 7 or 28 dpi. The levels of tumour necrosis factor-α (TNF-α), interleukin-1β (IL-1β), neuron-specific enolase (NSE) and astrocytic S100 calcium binding protein B (S100B) in the serum of the mice were measured via ELISA at 1 dpi.

#### In vitro

Neuro-2a cells or BV2 cells were cultured in RMPI-1640 medium supplemented with 10% fetal bovine serum. The cells were then treated as described below.

In neuro-2a cells, the ultrastructure, cell apoptosis and expression of the endoplasmic reticulum stress (ERS) apoptotic pathway were observed by transmission electron microscopy, annexin V-FITC and WB assays after they were treated with different doses of eCIRP for 48 h. Next, Neuro-2a cells were treated with 1 μg/ml eCIRP for 48 h after they were pretreated with GSK2656157 (0.5–2 μM) for 1 h. Then, the apoptotic pathway and apoptotic ratio were detected by WB and annexin V-Fluorescein isothiocyanate (FITC) assays.

In BV2 cells, the expression of inflammatory pathway components was detected by WB after they were treated with 1 μg/ml lipopolysaccharide (LPS) for 24 h after transfection with siCIRP or NC. In addition, BV2 cells were treated with eCIRP at different doses for 48 h or treated with 1 μg/ml eCIRP for 48 h after pretreatment with TAK-242 (1 μM) for 30 min or with MC1742 (1 μM) for 12 h. Then the release of inflammatory cytokines in BV2 cells was examined via ELISA.

### FPI

In this study, an animal model was generated using the hydraulic shock method, as shown in supplementary Figure 2b, see online supplementary material. The head was fixed to the stereotactic frame while the mouse was in the prone position. The skin was disinfected with iodine volt and alcohol. The scalp was cut along the midline to separate the periosteum and expose the skull. The structure of the brain was mapped by mouse stereotaxy using a brain stereoscopic positioning map. After sufficient drilling, a 3-mm-long window was created on the left parietal skull bone. A custom-made strike tube was tightly fixed to the nape around the bone window using reinforced dental zinc phosphate cement. Then the strike tube was filled with normal saline. After confirming that there was no leakage in the percussion tube or the percussion sleeve, the percussion tube was closely connected to the brain-trauma instrument and the pendulum height of the hydraulic percussion instrument was adjusted to ensure a stable percussion force. The mice were injured by hydraulic percussion. In the present study, the pendulum angle of the FPI device was varied between 9.8 and 10.8 degrees to bring about a peak pressure of between 1.1 and 1.3 atm when triggered against capped intravenous tubing. A Tektronix digital oscilloscope (TDS460A, Tektronix, Inc., Beaverton, OR, USA) was connected to examine the duration and peak pressure of the fluid pulse. The mice in the sham group were connected to the FPI device at 0°C. After injury, the mice were placed on their backs and their righting time was measured as an indicator of injury severity. After righting, the mice were re-anaesthetized, and the tube was removed. Finally, the scalp was disinfected and sutured and the animals were placed in a heated cage until they recovered. To choose the moderate-to-severe TBI model, FPI mice were included only if the righting reflex was >5 min according to the criteria of previous studies [21–23]. Details of the animal experiment are shown in supplementary Figure 2a, b.

At different time points after FPI, the mice were euthanized and the brains were removed. The tissues were collected from the damaged side of the brain in various regions (hippocampus or parietal cortex). Brain tissues from the same position from sham mice were dissected and used as controls. The tissues were frozen at −70°C for RNA or protein extraction.

### Real-time PCR

Briefly, total RNA was extracted with TRIzol reagent and reverse transcribed into cDNA with SuperScript III reverse transcriptase. Target gene expression was quantified. Glyceraldehyde 3-phosphate dehydrogenase was used as an internal control. The expression levels were calculated with the primer pairs used for the amplification of target mRNAs, as shown in Table 2. The data were analysed using the comparative cycle threshold method.

**Table 2:** Sequences of primers

Primer	Sequence (5' to 3')
GAPDH-F	AGGTCGGTGTGAACGGATTTG
GAPDH-R	TGTAGACCATGTAGTTGAGGTCA
CIRP-F	GGACTCAGCTTCGACACCAAC
CIRP-R	ATGGCGTCCTTAGCGTCATC
TNF-α-F	CCCTCACACTCAGATCATCTTCT
TNF-α-R	GCTACGACGTGGGCTACAG
IL-1β-F	GCAACTGTTCCTGAACTCAACT
IL-1β-R	ATCTTTTGGGGTCCGTCAACT

*GAPDH* glyceraldehyde 3 phosphate dehydrogenase, *CIRP* cold-inducible RNA-binding protein, *IL-1β* interleukin-1β, *TNF-α* tumor necrosis factor-α

### Western blotting

Cells or brain tissues were washed with phosphate buffer saline and treated with cell lysate. The mixture was centrifuged at 12,000 × g at 4°C for 10 min. The supernatant was collected for protein extraction using the Qproteome Mammalian Protein Prer Kit. The protein concentration was determined using a bicinchoninic acid protein assay kit. Then, 30 μg of protein was separated by polyacrylamide gel electrophoresis and transferred onto a nitrocellulose membrane by iBlot. Binds were visualized by chemiluminescence using a FluorChem E system. Antibodies against CIRP, GRP78, ATF4, CD206, caspase-3, cleaved caspase-3, Bcl-2, Bax, PERK and p-PERK were used to determine the expression levels of the proteins.

The immunoblot results were subsequently analysed using Image-Pro Plus 6.0 software to measure the area and grey value for each target band. The target protein and the internal reference were compared for semiquantitative analysis. Protein content = area of the band × average grayscale; semiquantitative target protein content = target protein content/glyceraldehyde 3-phosphate dehydrogenase protein content. All the data are presented as the mean ± SD.

### HE and immunofluorescence staining

The brain tissues were isolated on different days after TBI. The tissues were immersed in 4% paraformaldehyde overnight, embedded in paraffin and cut into sections (4 μm). The slices were dewaxed with xylene and washed with ethanol. The sections were stained with HE to assess lesion volume. The area of the lesion hemispheres was measured by ImageJ. The percent loss in volume was calculated by comparing the damaged area to the uninjured hemisphere as previously reported [21]. For immunofluorescence staining, the brain sections were treated with citric acid antigen repair liquid for 15 min at 96°C, immersed in 0.5% Triton X-100 for 30 min and blocked in 0.5% bovine serum albumin for 1 h at room temperature. Primary antibodies against GFAP, Iba-1, CIRP, CD86, CD206 and NeuN were added to the slides, which were then incubated at 4°C overnight. The next day, the slides were washed, incubated with secondary antibodies conjugated to Cy3 or Cy5 for 1 h and then with 4′,6-diamidino-2-phenylindole for 3–5 min. Approximately 8–10 brain sections were obtained from the damaged region. Positive cells in 5 brain sections were counted. The number of positive cells in the field of interest or the mean fluorescence intensity were assessed using Image-Pro Plus 6.0 software. Briefly, the optical density was calibrated and the area of interest was determined. Thereafter, the sum density of the integrated optical density was measured to calculate the mean density as previously reported [24,25].

### TUNEL assays

TUNEL assays were performed according to the instructions of the manufacturers. Briefly, the slides were rehydrated, proteinase K (20 mg/ml) was added, the slides were washed with PBS and refixed in 4% paraformaldehyde. The nucleotide mixture and Recombinant Terminal Deoxynucleotidyl Transferase enzyme were added to the slides at 37°C for 1 h and 2×saline sodium citrate was used to stop the reaction. The percentage of apoptotic cells was calculated as the number of positive cells/the number of all cells in at least four microscopic fields.

### Behavioural evaluation

**Mouse neurological severity score (mNSS)** An NSS of 10 points was used to estimate neurological injury after TBI, and the motor, reflex and balance abilities of the animals were tested after nerve injury. A score of 0 to 10 represented the injury grade from no damage to severe injury. One or zero points were given for failed or successful tasks, respectively. Testing was performed by the investigators who were blinded to the animal groups. In the present study, NSSs were established at 4 and 24 h, and on dpi 1, 3, 7, 21 and 28.

**Open field test** Anxiety-like behaviour and locomotor activity after nerve injury were assessed in the open as previously described. Briefly, mice were placed in an empty arena (45 × 45 × 30 cm) on dpi 7, 14, 21 and 28. The mice were allowed to freely explore for 5 min. Movement trials were recorded with an overhead camera. The time or distance in the center of the arena (29.5 × 29.5 cm) and the number of entries in the centre were calculated using EthoVision XT version 9 software (Noldus Information Technology, Leesburg, VA, USA).

**Y-maze test** Exploratory activity and memory ability were examined using a Y-maze test (Y-maze parameter, arm length 40 cm; upper and lower arm width 13 cm or 3 cm; wall height 15 cm; BrainScience Idea, Osaka, Japan). Mice were placed in the central area, and the time and number of trips that each mouse spent in each arm were recorded using the EthoVision XT video imaging system within 10 min.

## ELISA

The levels of eCIRP, TNF-α, IL-1β, NSE and S100B in the serum or cell supernatants were measured via ELISA kits, according to the manufacturer’s protocols.

### Nonlabelled quantitative acetylation of proteomics

The tissues in the damaged region of the WT or KO mice were collected at 1 dpi and subsequently sent to Shanghai GeneChem Co. for proteomic analysis (Shanghai, China). Briefly, the proteins were extracted and quantified from the samples. The proteins were subsequently separated and digested. The resulting peptides were collected and subjected to Kac enrichment with an acetyl-lysine motif (Ac-K) kit. The samples were analysed on a nanoElute coupled to a timsTOF Pro (Bruker, Bremen, Germany) instrument equipped with a CaptiveSpray source. The mass spectrum data were analysed using MaxQuant software version 1.6.14.0. Lysine acetylation sites with a fold change > 2 or < 0.5 and a *p* value (Student’s t test) < 0.05 were considered differential acetylation sites.

### Transmission electron microscopy

Cells from different groups were fixed in 2.5% glutaraldehyde and photographed by a JEOL JEM 1210 transmission electron microscope (JEOL, Peabody, MA, USA) at 80 or 60 kV on a thin-film369 electron microscope (ESTAR thick base; Kodak, Rochester, NY, USA).

### Statistical analysis

The data are shown as the mean ± standard deviation of the mean (SD). GraphPad Prism 9 was used for statistical analyses. The normality of the distribution was determined using the Shapiro–Wilk test. For the experiments involving two groups, an unpaired t test was used to assess normally distributed data, and the Mann–Whitney U test was used to assess nonnormally distributed data. One- or two-way analysis of variance was used to analyse significant differences among the groups, which was followed by Tukey’s test for *post hoc* multiple comparisons if significant effects existed in the main interaction. Outlier data were identified by GraphPad Prism 9 and removed. Spearman correlation analysis was used to evaluate the correlation between nonnormally distributed data. Pearson correlation analysis was used for normally distributed data. *P* values < 0.05 were regarded as statistically significant.

## Results

### Establishment of the TBI model

To investigate the potential role of CIRP in the pathogenesis of TBI, WT and neural-specific CIRP KO TBI mice were subjected to lateral FPI. After FPI, the mortality rate was ~7.8% in WT TBI mice and 7.1% in KO TBI mice at 24 h. Approximately 80% of all TBI mice exhibited seizure activity immediately after FPI. It is well known that a righting time >5 min indicates moderate-to-severe TBI, and the average righting time was 530 ± 36 s in this study (data not shown).

### CIRP expression is upregulated in TBI animals

**Figure 1:**
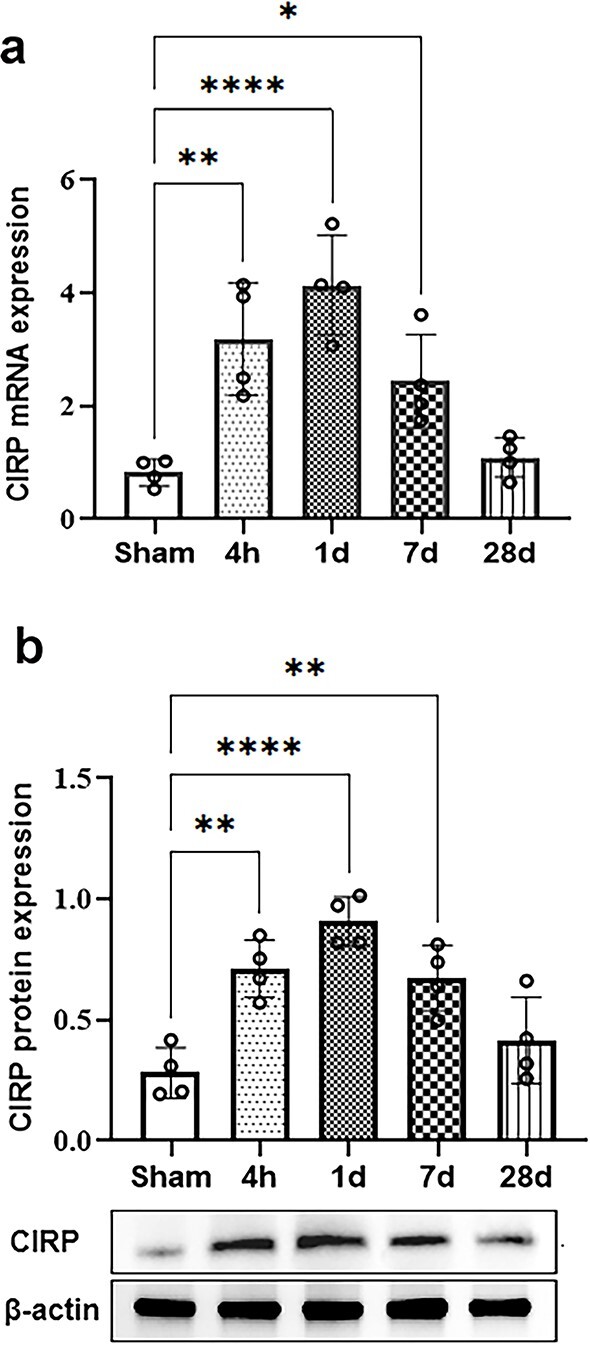
CIRP is abnormally expressed during the development of TBI. (**a**, **b**) CIRP expression in the damaged region of the brain of TBI mice was measured by q-PCR and WB at different time points, and GAPDH was used as the internal standard (n = 4). The data are expressed as the mean ± SD. Statistical significance: ^*^p < 0.05; ^*^^*^p < 0.01; ^*^^*^^*^^*^p < 0.0001. *CIRP* cold-inducible RNA-binding protein, *GAPDH g*lyceraldehyde 3-phosphate dehydrogenase, *q-PCR* real-time quantitative polymerase chain reaction, *TBI* traumatic brain injury, *WB* Western blot

First, we measured the expression levels of CIRP in the damaged brain regions at different time points after TBI. The results of q-PCR analysis showed that the mRNA expression of CIRP increased at 4 h, peaked on dpi 1, and gradually decreased from 7 to 28 dpi (3.1-, 4.1-, and 2.4-fold increases in TBI mice vs. sham mice at 4 h and 1 and 7 dpi, respectively; all *p* < 0.05; Figure 1a). Similarly, the results of a WB assay revealed that CIRP protein expression peaked at 1 dpi and remained high for at least 7 days after TBI (Figure 1b).

### CIRP deficiency alleviates injury volume and neuronal apoptosis after TBI *in vivo*

**Figure 2:**
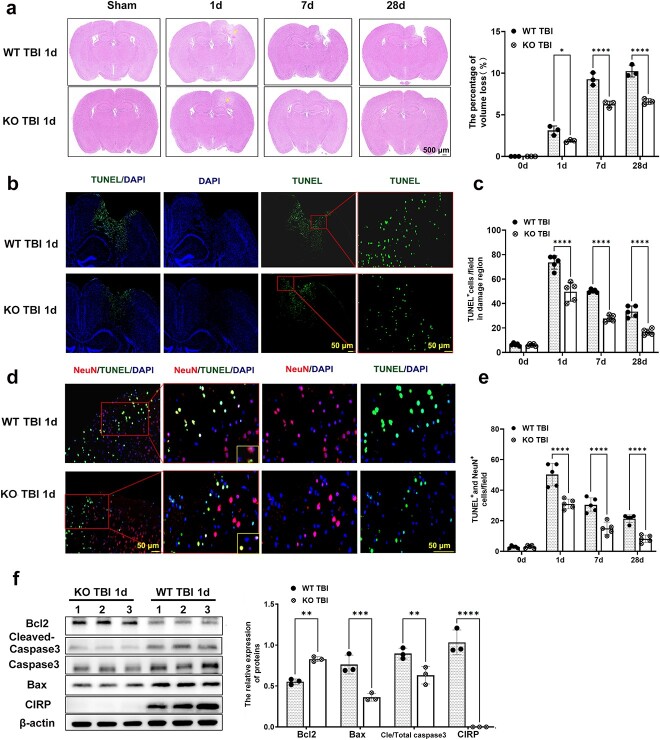
CIRP knockout alleviates brain injury and cell apoptosis after TBI *in vivo.* (**a**) Tissue loss was recorded by HE-stained images and quantified by ImageJ software on different days after TBI. The degree of tissue loss was calculated as the area of the region of loss compared to the area of the undamaged hemisphere (n = 3, scale bars: 500 µm). (**b**) TUNEL-stained (TUNEL^+^: TUNEL positive) images showing cell apoptosis in the damaged region of the brain from WT and CIRP KO TBI mice at 1 dpi (scale bar:  50 μm). (**c**) Histograms showing cell apoptosis in the various groups at different time points after TBI (n = 5). (**d**) Images showing neuronal cell apoptosis (TUNEL^+^/NeuN^+^: TUNEL positive/ NeuN positive) in regions of the brain damaged by TBI in WT or KO mice at 1 dpi (scale bars: 50 or 20 μm). (**e**) Histograms showing the analysis of neuronal apoptosis at different time points after TBI (n = 5). (**f**) CIRP, Bcl-2, caspase-3, cleaved caspase-3 and Bax expression in damaged regions of the brain from WT or KO TBI mice was examined via WB analysis at 1 dpi (n = 3). The data are presented as the mean ± SD. Statistical significance: ^*^p < 0.05; ^*^^*^p < 0.01; ^*^^*^^*^p < 0.001; ^*^^*^^*^^*^p < 0.0001. *BAX* Bcl-2 associated X protein, *Bcl-2* B cell lymphoma gene-2, *CIRP* cold-inducible RNA-binding protein, *TBI* traumatic brain injury, *d* day, *dpi* day post-traumatic brain injury, *HE* hematoxylin and eosin, *KO* neural-specific CIRP knockout, *NeuN* neuronal nuclei, *TUNEL* TdT mediated dUTP nick-end labelling, *WB* Western blot, *WT* wild type

Next, cortical tissue loss was estimated using HE staining at 1, 7 and 28 dpi. As shown in Figure 2a, the hemispheric volume loss was ~1.86, 6.25 and 6.59% in the KO TBI mice, and 3.11, 9.27 and 10.24% in the WT TBI mice at 1, 7 and 28 dpi, respectively. There were significant differences between the WT and KO TBI mice at various time points. The hemispheric volume loss in the WT TBI mice was ~167% greater at 1 dpi, 148% greater at 7 dpi and 155% greater at 28 dpi than that in the KO TBI mice (interaction F [3,16] = 20.21; group effect F [1,16] = 114, all *p* < 0.001; Figure 2a). Then, cell apoptosis in the brain was examined by TUNEL assay at 1, 7 and 28 dpi (Figure 2b, and supplementary Figure 3a, see online supplementary material). As shown in Figure 2b, a large quantity of TUNEL^+^ cells was observed in the damaged region of the TBI mice at 1 dpi. There were 148, 180 and 200% more TUNEL-positive cells in the WT TBI mice than in the KO TBI mice at 1, 7 and 28 dpi, respectively (interaction F [3,32] = 18.48; group effect F [1,32] = 160.6; all *p* < 0.001; Figure 2c).

The apoptosis of neurons in the damaged region was examined by TUNEL assay and immunofluorescence staining for NeuN (a specific marker of neurons) at various time points (Figure 2d and supplementary Figure 3b). TUNEL^+^/NeuN^+^ cells were 162, 202 and 261% more common in WT TBI mice than in KO TBI mice at 1, 7 and 28 dpi, respectively (interaction F [3,32] = 12.36; group effect F [1,32] = 98.34; all *p* < 0.001; Figure 2e). Furthermore, we examined the level of apoptotic proteins in the damaged region of the brain at 1 dpi using a WB assay. The results showed that the expression of Bax and cleaved caspase-3 was obviously downregulated. In contrast, the expression of Bcl-2 was upregulated in the KO TBI group compared to the WT TBI group (interaction F [3,16] = 79.77; group effect F [1,16] = 157.7; *p* < 0.001; Figure 2f).

**Figure 3:**
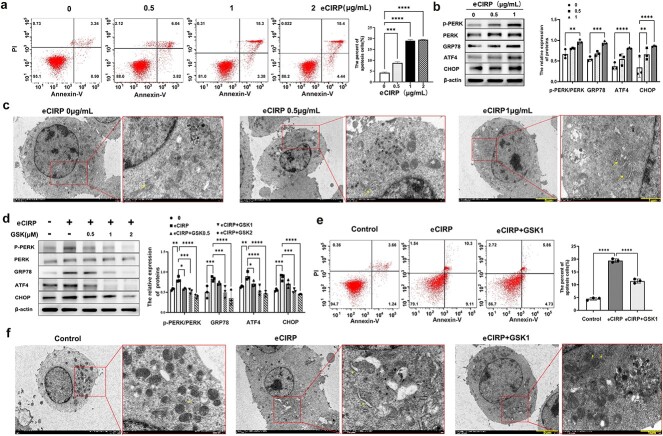
eCIRP regulates neuronal cell apoptosis via the ERS pathway. Neuro-2a cells were cultured with eCIRP at different concentrations (0.5, 1 and 2 μg/ml) for 48 h. Cells cultured without eCIRP for 48 h were used as controls. (**a**) Apoptotic rates of neuro-2a cells were analysed via flow cytometry after treatment with different doses of eCIRP (n = 3). (**b**) Expression levels of GRP78, p-PERK, PERK, CHOP and ATF4 in neuro-2a cells from the different groups were determined via WB (n = 3). (**c**) Ultrastructural alterations in neuro-2a cells were observed via electron microscopy after treatment with different doses of eCIRP. The arrows indicate the ER (scale bars: 5 or 1 μm). (**d**) Cells were pretreated with various concentrations of GSK2656157 for 1 h and then stimulated with eCIRP (1 μg/ml) for 48 h. Untreated cells were cultured for 48 h as controls. Expression levels of GRP78, PERK, p-PERK, CHOP and ATF4 in neuro-2a cells in the different groups were examined via WB. (**e**) Cells were pretreated with GSK2656157 (1 μg/ml) for 1 h and stimulated with eCIRP (1 μg/ml) for 48 h. Untreated cells were cultured for 48 h as controls. The apoptotic rates of neuro-2a cells in different groups were analysed via flow cytometry. (**f**) Ultrastructural changes in neuro-2a cells in various groups were analysed via electron microscopy. The arrows indicate the ER (scale bars: 5 or 1 μm). The data are presented as the mean ± SD from three independent experiments (n = 3). Statistical significance: ^*^p < 0.05; ^*^^*^p < 0.01; ^*^^*^^*^p < 0.001; ^*^^*^^*^^*^p < 0.0001. *ATF4* transcription factor 4, *CHOP* C/EBP homologous protein, *eCIRP* extracellular cold-inducible RNA-binding protein, *ER* endoplasmic reticulum, *ERS* endoplasmic reticulum stress, *GRP78* glucose-regulated protein 78, *GSK2656157* inhibitor of protein kinase RNA-like ER kinase, *PERK* protein kinase RNA-like ER kinase, *PI* Propidium Iodide, *p-PERK* cleaved protein kinase RNA-like ER kinase, *WB* Western blot

**eCIRP induces neuronal cell apoptosis via the ERS-related apoptotic pathway** Cells were cultured with different doses of eCIRP (0, 0.5, 1 and 2 μg/ml) for 48 h. As shown in Figure 3a, treatment with eCIRP *in vitro* resulted in cell apoptosis, especially at a dose of 1 μg/ml. However, there were no obvious differences in the apoptosis of neurons between the 1 and 2 μg/ml groups. Thus, we examined the ultrastructure and expression of ERS markers including p-PERK, GRP78, ATF4 and CHOP in neuron-2a cells after they were treated with 0, 0.5 or 1 μg/ml eCIRP for 48 h. The results confirmed that eCIRP treatment, especially at a dose of 1 μg/ml, could activate apoptosis pathways related to ERS in neuron-2a cells and upregulate the expression of p-PERK, GRP78, ATF4 and CHOP in neuron-2a cells (interaction F [6,24] = 0.41, *p* = 0.861; group F [2,24] = 51.35; *p* < 0.001, Figure 3b) and cause significant expansion of the ER (Figure 3c).

Next, neuron-2a cells were pretreated with GSK2656157 (an inhibitor of PERK) at different doses (0.5–2 μmol/l) for 1 h, and then exposed to 1 μg/ml eCIRP for 48 h to further investigate whether eCIRP could induce neuronal cell death through the PERK–ATF4–CHOP pathway, a critical ERS-related apoptosis pathway. As shown in Figure 3d, treatment with GSK2656157 significantly diminished the eCIRP-induced increase in the expression of ERS-related apoptotic proteins in a dose-dependent manner. In addition, 1 μmol/l GSK2656157 could almost completely abolish the alterations in PERK, ATF4, CHOP and GRP78 expression. (interaction F [12,40] = 0.5347, *p* = 0.8789; group F [4,40] = 43.54, *p* < 0.001; Figure 3d) Moreover, inhibition of the PERK signalling pathway with 1 μmol/l GSK2656157 alleviated eCIRP-induced apoptosis and ER expansion in neuron-2a cells (Figure 3e, f).

### CIRP deficiency inhibits astroglial and microglial responses after TBI

Herein, we assessed glial activation by immunofluorescence staining for GFAP or Iba-1 (specific makers of astroglia or microglia) in damaged brain regions of WT or KO TBI mice at different time points after TBI.

**Figure 4:**
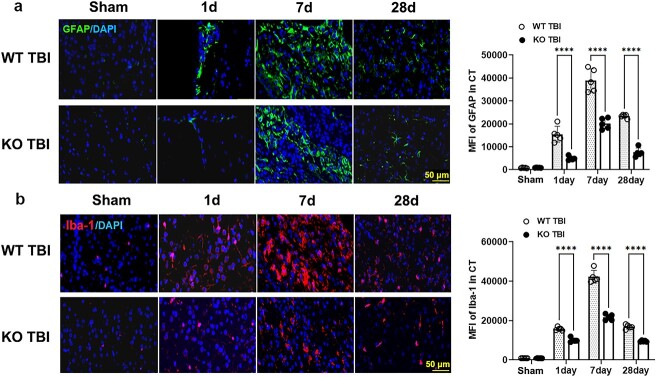
CIRP knockout inhibits the activation of microglia/astrocytes after TBI. (**a**) Images and histograms of astrocyte activation. Immunofluorescence staining and statistical analysis of GFAP expression in the damaged cortex of WT or KO TBI mice at different time points after TBI (scale bar : 50 μm). (**b**) Images and histograms of microglial activation. Immunofluorescence staining and statistical analysis of Iba-1 expression in damaged cortex from WT or KO TBI mice at different time-points after TBI (scale bar: 50 μm). The data are presented as the mean ± SD from five independent experiments; ^*^^*^^*^^*^p < 0.0001. *DAPI* 4′,6-Diamidino-2-phenylindole, *CIRP* cold-inducible RNA-binding protein, *d* day, *GFAP* glial fibrillary acidic protein, *Iba-1* ionized calcium binding adapter molecule-1, *KO* neural-specific CIRP knock-out, *MFI* mean fluorescence intensity, *TBI* traumatic brain injury, *WT* wild-type

GFAP staining revealed that astrogliosis occurred after TBI, as indicated by the increase in the percentage of GFAP-positive cells in the damaged region of the brain. Astrocytes became hypertrophic, with a significant overlap of astroglial protrusions in TBI mice from 1 to 7 dpi (supplementary Figure 4a, see online supplementary material). Notably, the fluorescence intensity of GFAP in the damaged cortex of WT TBI mice was much greater than that in the damaged cortex of KO TBI mice. Approximately 3.10-, 1.93- and 3.09-fold increases were observed in the WT TBI mice vs. the KO TBI mice at 1, 7 and 28 dpi, respectively (interaction F [3,32] = 95.74; group effect F [1,32] = 356.9; all *p* < 0.001; Figure 4a).

Iba-1 staining revealed that the number of microglia in the cortex increased at 1 dpi, accumulated continuously at 7 dpi, and decreased at 28 dpi in both WT and KO TBI mice. With regard to cell morphology, the morphology of the microglia changed from a branching shape to a retractable, relatively large cellular body and macrophage-like shape from 1 to 7 dpi, and most of the microglia developed a branching shape at 28 dpi (supplementary Figure 4b). In addition, the fluorescence intensities of Iba-1 in the damaged cortex of WT mice were ~1.51, 2.12 and 1.76 times greater than those in KO TBI mice at 1, 7 and 28 dpi, respectively (interaction F [3,32] = 28.84; group effect F [1,32] = 211.6; *p* < 0.001; Figure 4b).

### CIRP deficiency inhibits the M1 polarization of microglia after TBI

**Figure 5:**
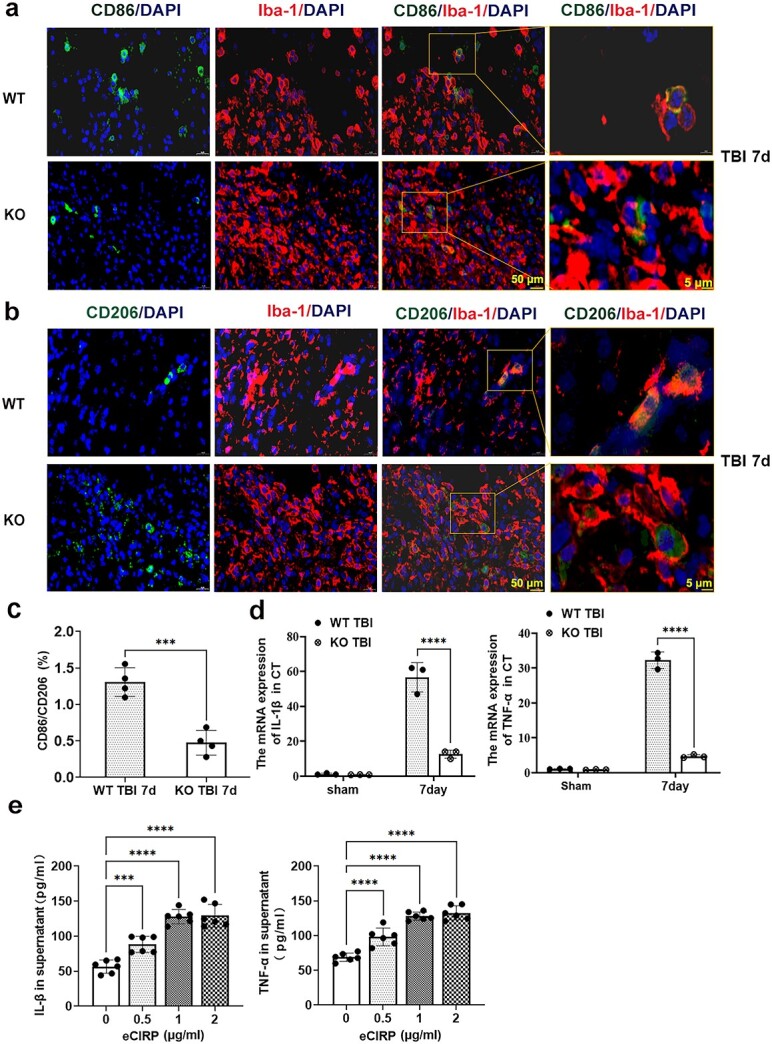
Impact of CIRP on microglial polarization *in vivo* and *in vitro*. Images of M1 (**a**) or M2 (**b**) differentiation of microglia in the damaged cortex were examined by immunofluorescence staining for Iba-1/CD86 or Iba-1/CD206 at 7 dpi (scale bars:  50 or 5 μm). (**c**) Statistical analysis of the Iba-1/CD86 and Iba-1/CD 206 ratios in the damaged cortex of KO or WT TBI mice at 7 dpi, n = 3. (**d**) TNF-α and IL-1β mRNA expression in the damaged cortex of KO or WT TBI mice was measured by q-PCR at 7 dpi. β-Actin served as the internal standard and WT sham mice were used as the controls (n = 3). (**e**) TNF-α and IL-1β levels in the supernatants of BV2 cells. BV2 cells were cultured with eCIRP at different concentrations (0.5, 1 and 2 μg/ml) for 48 h. Cells cultured without eCIRP for 48 h were used as controls. Levels of TNF-α and IL-1β in the supernatants of BV2 cells were measured via ELISA after CIRP treatment; n = 6. The data are expressed as the mean ± SD; ^*^^*^^*^p < 0.001; ^*^^*^^*^^*^p < 0.0001. *DAPI* 4′,6-Diamidino-2-phenylindole, *d* day, *dpi* day post-traumatic brain injury, *CT* damaged cortex, *eCIRP* extracellular cold-inducible RNA-binding protein, *ELISA* enzyme-linked immunoassay, *Iba-1* ionized calcium binding adapter molecule-1, *IL-1β* interleukin-1β, *KO* neural-specific CIRP knockout, *M1* M1-like phenotype of microglia, *M2* M2-like phenotype of microglia, *q-PCR* real-time quantitative polymerase chain reaction, *TBI* traumatic brain injury, *TNF-α* tumor necrosis factor-α, *WT* wild-type

We examined the M1/M2 polarization of microglia by immunofluorescence staining for Iba-1/CD86 (Figure 5a) or Iba-1/CD206 (Figure 5b) at 7 dpi. The results showed that the ratio of M1 (Iba-1^+^ and CD86^+^ cells)/M2 (Iba-1^+^ and CD206^+^ cells) cells in the damaged region was significantly lower in the KO TBI group than in the WT TBI group (0.4892 ± 0.2040 vs. 1.29 ± 0.2371, *p* < 0.01, Figure 5c). The mRNA expression levels of TNF-α and IL-1β in the damaged cortex of mice were also examined via q-PCR. Notably, both TNF-α and IL-1β mRNA expression levels were significantly greater in the TBI group than in the sham group. However, knockout of CIRP obviously downregulated TNF-α (interaction F [1,8] = 373.7; group effect F [1,8] = 385.8; all *p* < 0.001; Figure 5d) and IL-1β expression in the region damaged by dpi 7 (interaction F [1,8] = 75.29; group effect F [1,8] = 77.78; all *p* < 0.001; Figure 5d).

Similarly, we examined the potential impact of eCIRP on microglial polarization *in vitro*. The results showed that eCIRP treatment promoted the activation of BV2 cells towards the proinflammatory M1-like phenotype, as evidenced by the apparent increase in TNF-α and IL-1β secretion 48 h after eCIRP treatment in a dose-dependent manner compared with that in the untreated group (all *p* < 0.001; Figure 5e).

### CIRP deficiency alleviates neurobehavioural dysfunction after TBI

**Figure 6:**
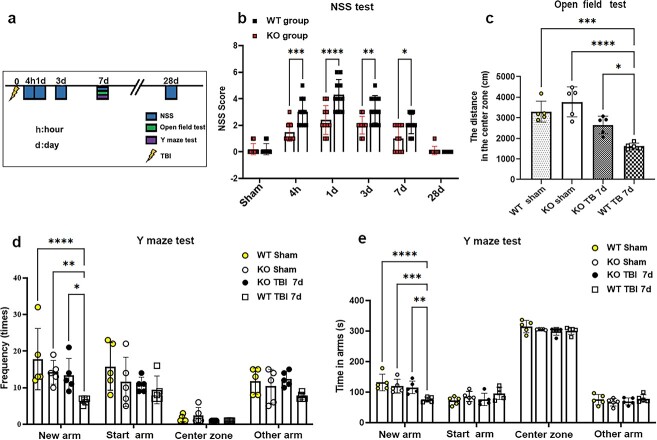
CIRP deficiency alleviates the behavioural deficits induced by TBI. (**a**) Experimental protocol. (**b**) NSS scores of WT and KO TBI mice were assessed at different intervals after TBI, and the data were analysed by two-way analysis of variance (n = 8). (**c**) Open field test. The distance in the centre zone of the mice was recorded at 7 dpi (n = 5). (**d**) The frequency of mice entering the different arms was recorded using the Y-maze test at 7 dpi (n = 5). (**e**) The time that mice spent in the different arms was recorded by the Y-maze test at 7 dpi (n = 6). The data are shown as mean ± SD. ^*^p < 0.05; ^*^^*^p < 0.01; ^*^^*^^*^p < 0.001; ^*^^*^^*^^*^p <0.0001. *CIRP* cold-inducible RNA-binding protein, *d* day, *dpi* day post traumatic brain injury, *KO* neural-specific CIRP knockout, *NSS* neurological severity score, *TBI* traumatic brain injury, *WT* wild-type

The neurological functions of the mice were evaluated using the 10-point mNSS test, the Y-maze test and the open field test at different time-points after TBI (Figure 6a). The results of the mNSS test revealed obvious neurological dysfunction in TBI mice at 4 h and at 1 and 3 dpi. Both KO and WT TBI mice tended to recover, with the median NSS improving to 1 and 2.1, respectively, at 7 dpi. However, at 4 h after TBI, the WT TBI mice exhibited greater dysfunction than did the KO TBI mice at 1, 3 and 7 dpi (interaction F[5108] = 5.26; group effect F[1108] = 41.75; all *p* < 0.001; Figure 6b).

The hippocampal memory abilities and locomotor activity of both KO and WT TBI mice were assessed using the open field test and the Y-maze test at 7 dpi. In the open field test, the KO TBI mice stayed in the central area longer than the WT TBI mice did (KO TBI-7d vs*.* WT TBI-7d = 2649 ± 193.7 s vs*.* 1618 ± 68.45 s, *p* < 0.01; Figure 6c), although the TBI mice travelled less distance into the central region than the sham mice did. However, there were no significant differences in locomotor speed among the four groups (data not shown). In the Y-maze test, KO TBI mice entered the novel arm more frequently than WT TBI mice did at 7 dpi (interaction F[9,64] = 1.434, *p* = 0.1928; group effect F[3,64] = 6.809, *p* < 0.001; Figure 6d). In addition, KO TBI mice spent more time in the novel arm than did WT TBI mice at dpi 7 (interaction F[9,64] = 4.278; group effect F[3,64] = 3.766; all *p* < 0.05; Figure 6d), which implied that mice in the KO TBI group were better able to explore new objects. Thus, the results of different behavioural experiments indicated that TBI might markedly result in cognitive dysfunction, while CIRP knockout could alleviate TBI-induced cognitive deficits and neurological damage.

### CIRP deficiency regulates histone H3 acetylation in the brain after TBI

**Figure 7:**
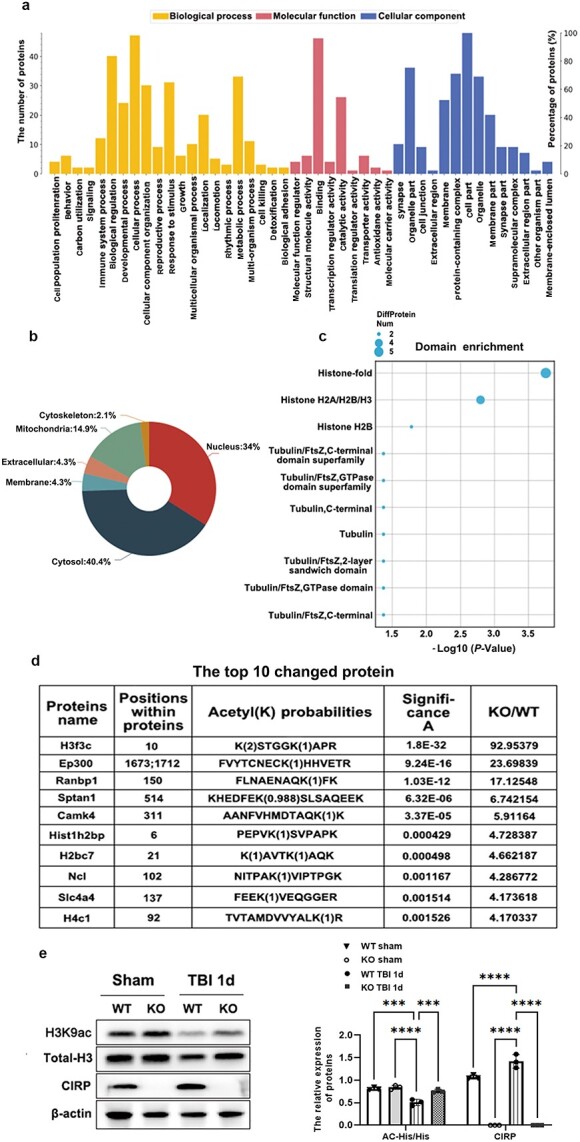
CIRP deficiency increased His acetylation at lysine 9 after TBI. Protein acetylation levels in the damaged cortex of WT or KO TBI mice were tested via label-free quantitative acetylation proteomics experiments at 1 dpi. (**a**–**c**) Histograms showing the functions, locations and domains of the significantly changed proteins at 1 dpi. (**d**) Table showing the top ten changed proteins and their acetylation positions. (**e**) Acetylation levels of histone H3 in the damaged cortex of WT or KO TBI mice were assessed via WB analysis at 1 dpi. The data are presented as the mean ± SD from three groups. ^*^^*^^*^p < 0.001; ^*^^*^^*^^*^p <0.0001. *CIRP* cold-inducible RNA-binding protein, *d* day, *dpi* day post-traumatic brain injury, *H3* histone H3, *H3K9ac* histone H3 acetylation levels at the lysine 9 site, *KO* neural-specific CIRP knockout, *TBI* traumatic brain injury, *WB* Western blot, *WT* wild-type

We further determined the changes in protein acetylation levels in the cerebral cortex of KO and WT TBI mice via label-free quantitative acetylation proteomics at 1 dpi. The acetylation levels of 58 proteins were obviously different between the two groups. These proteins were mainly associated with behaviour, cell proliferation and the immune response (Figure 7a). Most of these proteins were located in the nucleus and cytoplasm, as shown in Figure 7b. The results of the protein-based domain analysis revealed that most of the alterations in the protein domain were focused on the histone and tubulin domain superfamilies (Figure 7c). As presented in Figure 7d, histone H3 was one of the most significantly altered proteins according to acetylation level, and the possible acetylation site was at lysine 9.

To verify the results of proteomic experiments, we measured the levels of acetylated histone H3 at the lysine 9 site (H3K9ac) in the cerebral cortex of WT and KO TBI mice using a WB assay at 1 dpi. Histone H3 acetylation was decreased in the cerebral cortex of TBI mice compared to that in the sham group. In addition, the level of H3K9ac in the KO TBI group was significantly greater than that in the WT TBI group at 1 dpi (interaction F[9,64] = 4.278; group effect F[3,64] = 3.766; all *p* < 0.001; Figure 7e).

### CIRP mediates neuroinflammation by modulating histone H3 acetylation

**Figure 8:**
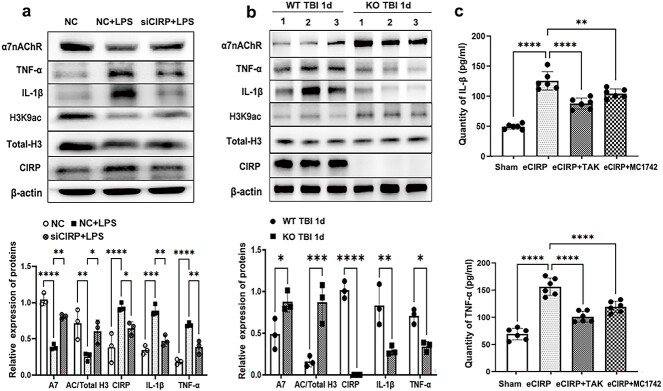
CIRP can mediate neuroinflammation by regulating histone H3 acetylation. (**a**) BV2 cells were treated with 1 μg/ml LPS for 24 h after they were transfected with CIRP-siRNA or NC, and the untreated BV2 cells that were transfected with NC were regarded as the controls. The expression levels of H3K9ac, α7nAChR, IL-1β and TNF-α in BV2 cells from different groups were examined via WB (n = 3). (**b**) H3K9ac, α7nAChR, IL-1β and TNF-α expression in the damaged cortex of WT and KO TBI mice was determined via WB at 1 dpi (n = 3). (**c**) BV2 cells were stimulated with 1 μg/ml CIRP protein for 48 h after MC1742 or TAK treatment, after which the IL-1β and TNF-α levels in the culture medium of the BV2 cells were measured via ELISA (n = 5). The data are presented as the mean ± SD. ^*^p < 0.05; ^*^^*^p < 0.01; ^*^^*^^*^p < 0.001; ^*^^*^^*^^*^p < 0.0001. *AC* acetylation, *α7nAChR* α7 nicotinic acetylcholine receptor, *CIRP* cold-inducible RNA-binding protein, *d* day, *dpi* day post-traumatic brain injury, *eCIRP* extracellular cold-inducible RNA-binding protein, *ELISA* enzyme-linked immunoassay, *H3* histone3, *H3K9ac* histone H3 acetylation levels at the lysine 9 site, *IL-1β* interleukin-1β, *KO* neural-specific CIRP knockout, *LPS* lipopolysaccharide, *MC1742* activator of histone H3 acetylation levels at the lysine 9 site, *NC* negative control, *TAK* inhibitor of toll-like receptor 4, *TBI* traumatic brain injury, *TNF-α* tumor necrosis factor-α, *WB* Western blot, *WT* wild-type

A previous report revealed that histone H3 acetylation is involved in the microglial inflammatory response after TBI [26]. Herein, we investigated whether CIRP deficiency could affect the activity of inflammatory pathways in microglial cells by acetylation of H3 in vitro using LPS treated cell models. BV2 cells were transfected with CIRP-siRNA or NC, 24 h later they were treated with 1 μg/ml LPS for 24 h. The expression of H3K9ac in different cells was examined by the WB assay, and the untreated BV2 cells that underwent NC were served as the control. The results showed that CIRP knockdown in BV2 cells alleviated the decrease in H3K9ac and α7nAChR expressions, thus attenuating the enhanced expressions of L-1β as well as TNF-α induced by LPS stimulation (interaction F(8,30) = 21.32, *p* < 0.001; group effect F(2,30) = 3.335, *p* < 0.05; Fig. 8a).

The WB analysis of TBI animals revealed that the levels of IL-1β and TNF-α were decreased but that the expression of H3K9ac and the α7 nicotinic acetylcholine receptor (α7nAChR) was augmented in KO TBI mice compared to WT TBI mice at dpi1 (interaction F(4,20) = 38.52, *p* < 0.001; group effect F(1,20) = 10.44, *p* < 0.05; Fig. 8b).

To further explore whether CIRP mediates neuroinflammation through histone H3 acetylation, BV2 cells were stimulated with 1 μg/ml eCIRP protein for 48 h after pretreatment with MC1742 (activator of H3K9ac) or TAK-242 (inhibitor of Toll-like receptor 4 [TLR4]). The secretion of IL-1β and TNF-α in the different groups was examined via ELISA. As shown in Figure 8c, after CIRP treatment, the IL-1β level in the culture medium of BV2 cells increased from 49.7 ± 1.740 to 125.8 ± 6.205 pg/ml, while after TLR4 blockade or H3K9ac activation, it decreased to 88.05 ± 3.709 or 105 ± 3.134 pg/ml, respectively. Accordingly, TNF-α levels increased from 69.59 ± 4.309 to 156.8 ± 6.517 pg/ml after treatment with eCIRP, while they decreased to 101.5 ± 4.088 or 119.8 ± 4.21 pg/ml after treatment with an inhibitor of TLR4 or activator of H3K9ac, respectively.

### eCIRP levels are positively associated with inflammatory mediator and TBI biomarker levels

**Figure 9:**
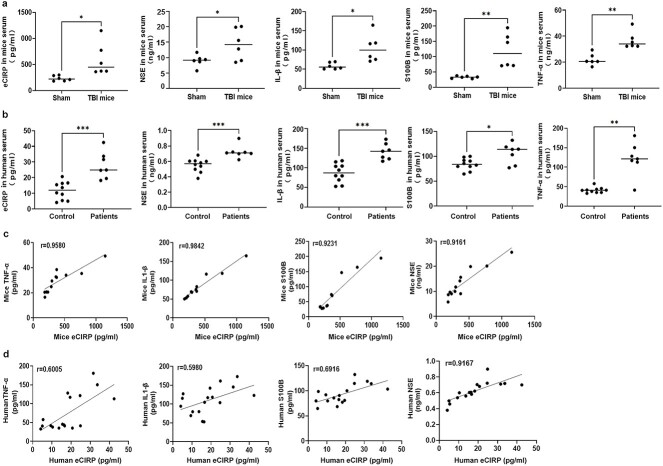
eCIRP levels are associated with inflammatory mediator and TBI biomarker levels. (**a**, **b**) eCIRP, TNF-α, IL-1β, NSE and S100β levels in serum from TBI mice or patients were measured via ELISA at 1 dpi (n = 6 in the TBI or sham mouse groups, n = 7 in the TBI patient group and n = 10 in the healthy control group). (**c**, **d**) The correlations between eCIRP and TNF-α, IL-1β, NSE, and S100β in mouse or human serum were analysed by Pearson’s method. The *x*-axis or *y*-axis represents the amount of eCIRP, TNF-α, IL-1β, NSE or S100β in the serum. The data are expressed as the mean ± SD. Statistical significance: ^*^p < 0.05; ^*^^*^p < 0.01; ^*^^*^^*^p < 0.001. *dpi* day post-traumatic brain injury, *eCIRP* extracellular cold-inducible RNA-binding protein, *ELISA* enzyme-linked immunoassay, *IL-1β* interleukin-1β, *NSE* neuron-specific enolase, *S100β* S100 calcium binding protein β, *TBI* traumatic brain injury, *TNF-α* tumor necrosis factor-α

Previous studies have suggested that there are close correlations between early increases in peripheral cytokines and poor outcomes in patients after TBI, and elevated levels of inflammatory cytokines, including TNF-α and IL-1β, are associated with unfavourable patient outcomes and death within 24 h of hospital admission [27,28]. Thus we explored the relationships between eCIRP and inflammatory mediators, including TNF-α and IL-1β, as well as between eCIRP and TBI biomarkers, including NSE and S100B, in serum from mice and humans at 1 dpi. The eCIRP levels were significantly elevated at 1 dpi in both mouse and human serum (all *p* < 0.05; Figure 9a, b); accordingly, the inflammatory mediator and biomarker levels in TBI mice and human serum were increased at 1 dpi compared with those in sham controls or healthy volunteers (all *p* < 0.05; Figure 9a, b). Moreover, at 1 dpi, there were positive correlations between eCIRP and TNF-α, IL-1β, NSE and S100B in both mice (eCIRP vs*.* TNF-α: *r* = 0.9580, *p* < 0.0001; eCIRP vs*.* IL-1β: *r* = 0.9842, *p* < 0.0001; eCIRP vs*.* NSE: *r* = 0.9231, *p* < 0.0001; eCIRP vs*.* S100B: *r* = 0.9161, *p* < 0.0001) and patients (eCIRP vs*.* TNF-α: *r* = 0.6005, *p* = 0.0027; eCIRP vs*.* IL-1β: *r* = 0.5980, *p* = 0.0028; eCIRP vs*.* NSE: *r* = 0.9167, *p* < 0.0001; eCIRP vs*.* S100B: *r* = 0.6916, *p* = 0.0004) (Figure 9c, d).

## Discussion

Intracellular CIRP is critically involved in cellular stress responses such as ultravioletr irradiation, hypoxia and hypothermia, and is able to protect cells from environmental changes. eCIRP is regarded as a damage-associated molecule in specific cells, including lymphocytes, epithelial cells, endothelial cells, neutrophils and microglia. Moreover, eCIRP is an essential mediator of the cerebral inflammatory response and can enhance the release of multiple inflammatory cytokines in ischaemia, trauma and alcoholism [29,30]. However, the potential functions and underlying mechanism of eCIRP in the pathogenesis of TBI remain to be further elucidated.

Herein, the relationship between CIRP expression and TBI-induced neuroinflammation was investigated through both *in vivo* animal models and *in vitro* cell experiments*.* We observed blood deposition and significant tissue damage (tissue colouration was different from that at normal sites) in the damaged regions of the TBI mice at 1 dpi, indicating that the TBI model was successfully established in the present study. First, we examined the location and quantity of CIRP in neural cells and blood. CIRP was found to be located mainly in the nucleus of normal neural cells; it translocated from the nucleus to the cytoplasm at 1 dpi (supplementary Figure 4c) and maintained a high level at least 7 days after TBI (Figure 1b). Moreover, the increased serum levels of CIRP were positively correlated with inflammatory mediator levels in both TBI animals and patients at 1 dpi. Previous reports have shown that CIRP is translocated during hypoxia and inflammation and that the administration of eCIRP to healthy rats could increase the serum inflammatory cytokines [31]. Our results are in agreement with those of previous studies and confirm the close relationship between eCIRP and the inflammatory response.

The development of TBI is a complex pathophysiological process. Therefore, identifying reliable biomarkers in biological fluids, including blood and cerebrospinal fluid, is highly valuable. S100B and NSE are known to be neural-specific biochemical markers, and serum levels of NSE and S100B can reflect the severity of brain damage with high specificity and sensitivity [32]. In this study, we detected that there were positive correlations between CIRP and NSE or S100B, which suggested that CIRP might be used as a biomarker for assessing the severity and prognosis of TBI.

Increasing evidence has indicated that CIRP deficiency can relieve neural injury induced by DHCA, cardiopulmonary bypass, and alcohol-induced or hypoxic-ischaemic brain injury [16–18,20]. Therefore, we speculated that CIRP might also be involved in neuronal apoptosis following TBI. Currently, most studies investigating the role of CIRP in neurological inflammation involve systemic CIRP knockout mice. Loss of the peripheral CIRP gene may affect the progression of central inflammation. In contrast, the central conditional knockout model can overcome this shortcoming and better reflect the central regulatory effect of CIRP in mediating neuroinflammation. Thus, we established TBI animal models using neural CIRP gene-specific knockout mice to accurately explore the potential role and mechanism of CIRP in TBI-induced brain damage. CIRP deficiency occurs in both neurons and glial cell precursors in our animal models. The results showed that CIRP deficiency in the central nervous system obviously ameliorated the lesion volume and neuronal apoptosis in TBI animals. Moreover, CIRP deficiency negatively regulated Bax expression and enhanced Bcl-2 expression, thus decreasing caspase-3 activation in brain tissues after TBI. It is well accepted that Bcl-2 and Bax are important components of the apoptotic pathway that can inhibit or induce the expression of caspase-3, the conductor of apoptosis. Taken together, these data suggest that CIRP is a vital contributor to neuronal apoptosis.

Until recently, the specific mechanisms underlying eCIRP-induced neuronal cell apoptosis have not been fully understood. Many studies indicate that ERS is a pivotal factor in disrupting cellular stability and inducing apoptosis [33]. In the setting of TBI, persistent ERS induced by secondary cerebral injuries is considered a key contributor to cell apoptosis as well as uncontrolled neuroinflammation. For example, Shimizu *et al*. [34] reported that CIRP promoted inflammation and apoptosis in the lungs resulting from sepsis by triggering ERS. Therefore, we hypothesized that eCIRP released by neural cells might lead to neuronal cell apoptosis via the induction of ERS following TBI. In the present study, we observed that stimulation with eCIRP for 48 h markedly augmented neuronal apoptosis, as indicated by the apparent alteration and expansion of the ER. These results reveal, for the first time, a link between CIRP and ERS in the development of TBI.

When ERS is induced by continuous stimulation from a harmful environment, PERK dissociates from GRP78 and is autophosphorylated, thereby promoting the expression of ATF4 and CHOP. As a critical transcription factor of ERS-mediated cell death, CHOP is an important regulator of Bcl-2 family members, and persistent activation of CHOP can induce cell apoptosis [35,36]. Recently, Yi *et al*. [37] reported that PERK–ATF4–CHOP was associated with glucocorticoid-induced neuronal apoptosis. In a previous study, we found that high mobility group box-1 protein, a downstream cytokine of CIRP in inflammation, induced ERS-related apoptosis via the PERK–ATF4–CHOP pathway [38]. Herein, we revealed the activation of PERK as well as the elevation of GRP78, ATF4 and CHOP in neurons after eCIRP stimulation. Moreover, treatment with a PERK signalling inhibitor not only alleviated the activation of the PERK–ATF4–CHOP pathway but also inhibited the eCIRP-induced apoptosis and expansion of the ER. These results suggest that the excessive response to ERS evoked by a high concentration of eCIRP during TBI might contribute to neuronal apoptosis by activating the PERK–ATF4–CHOP signalling pathway.

Although several studies have shown that simple M1/M2 polarization cannot fully represent the functional heterogeneity of microglia [39,40], classifying M1/M2 microglia is still helpful for understanding the functional status of microglia in the pathogenesis of TBI [41]. It is well known that M1 microglia coexpress Iba-1 (a special microglial marker) and biomarkers such as CD86, CD16 and MHC-II, while the M2 microglia coexpress Iba-1 and phenotypic markers, including CD206, CD163 and Arg-1. The persistent M1-like polarization of microglia strongly affects functional outcomes during TBI; thus, regulating the balance of M1/M2 polarization in the microglia is crucial for the development of TBI [42–44]. Previous studies reported that recombinant eCIRP could boost the secretion of proinflammatory cytokines from murine microglia in a time- and dose-dependent manner. In contrast, inhibition of CIRP diminishes microglial activation in DHCA-induced rat brain injury [18,20]. In our animal models, CIRP deficiency occurred in both neurons and glial cell precursors; CIRP deficiency not only regulated neurons but also modulated microglia. We noted for the first time that knockout of CIRP in glial cell precursors inhibited the M1-like phenotype of microglia and therefore alleviated the functional deficiency of mice during TBI.

Acetylation is a kind of epigenetic modification that regulates gene expression independently of the DNA sequence. A series of studies indicated that protein acetylation is involved in mediating neuroinflammation following TBI. For instance, Gao *et al*. [45] noted that the acetylation level of histone H3 was significantly decreased in the brain from hours to days after TBI, which was associated with secondary brain pathology after TBI. However, microglial activation and neuronal degeneration evoked by TBI can be alleviated by increasing histone acetylation in the brain [26]. In the present study, we found that CIRP knockout markedly upregulated the acetylation level of histone H3 in the cerebral cortex after TBI at 1 dpi.

As the most prominent component of the cholinergic anti-inflammatory pathway, the α7nAChR is closely related to many neurological disorders including neuroinflammation, Parkinson’s disease and Alzheimer’s disease [46–48]. Recently, it was reported that TLR4 could inhibit the expression of α7nAChR through histone deacetylase activity in BV2 cells during neuroinflammation [48]. Notably, TLR4 is a cell surface receptor involved in eCIRP signalling [31]. Thus, it is reasonable for us to speculate that eCIRP may affect the α7nAChR by regulating the acetylation level of histones via TLR4. In the present study, the LPS-induced reductions in α7nAChR and H3K9ac levels were alleviated by CIRP knockdown. Consequently, the LPS-induced formation of proinflammatory mediators was inhibited by the downregulation of CIRP in BV2 cells. Similar results were obtained from *in vivo* experiments. Furthermore, we observed that pretreatment with an activator of acetylated histone H3 or with an inhibitor of TLR4 significantly inhibited the eCIRP-induced release of proinflammatory cytokines in BV2 cells. These findings suggest that CIRP may impact the microglial inflammatory response by regulating α7nAChR expression through histone modification via TLR4.

In addition to microglia, astrocytes play important roles in neuroprotection and inflammation. It is well accepted that astrocyte activation can mediate neuroinflammation by enhancing the release of inflammatory mediators secondary to TBI. Astrogliosis and astrogleneration are the main pathological changes in astrocytes during brain injury, as are the upregulation of GFAP expression [47,49]. Similarly, we noted hypertrophic astroglial cells with more robust expression of GFAP in the damaged regions of mice after TBI. However, CIRP deficiency significantly attenuated the activation of astrocytes following TBI.

Taken together, the results of our study confirmed the important role and significance of eCIRP in TBI and led to a deeper understanding of the molecular signalling pathway involved in neuroinflammation, in turn revealing a potential novel biomarker and therapeutic target for improving outcomes after TBI. Nevertheless, the current work must be interpreted in the context of a number of limitations. First, we did not explore the underlying mechanism by which CIRP regulates astrocyte activation during TBI. Second, we examined the effect of CIRP on histone H3 acetylation but did not provide a precise regulatory pathway linking CIRP and histone H3 acetylation. Third, we only observed the survival rates of TBI mice within 24 h after TBI due to the limited number of neural-specific CIRP knockout mice, and long-term survival rates should be recorded in our further studies. Fourth, we examined the expression of CIRP in brain tissues only after TBI *in vivo* and explored the molecular mechanism of TBI using neurons and glial cell lines *in vitro*. It is more reliable to perform these experiments with primary neurons and glial cells during TBI. Finally, TBI appears to be a key contributor to neurodegenerative disease, and a recent study revealed that eCIRP is involved in the development of Alzheimer’s disease [50], which suggested that eCIRP might be associated with TBI-induced neurodegenerative diseases. Therefore, additional clinical and basic studies are needed to further investigate the potential significance of CIRP in long-term brain dysfunction in the setting of TBI.

## Conclusions

In summary, this study demonstrated that eCIRP is induced by brain injury and exerts a harmful impact on the development of TBI by augmenting neuronal apoptosis via the ERS pathway and regulating M1/M2 polarization of microglia via the TLR4/histone H3/α7nAChR pathway. Therefore, the downregulation of eCIRP expression can significantly inhibit neuronal cell apoptosis and the activation of microglia/astrocytes and might serve as a key target for the intervention of uncontrolled neuroinflammation and subsequent brain dysfunction resulting from severe TBI.

## Abbreviations

α7nAChR: α7 Nicotinic acetylcholine receptor; AC-H3: Acetylated histone H3; ATF4: Transcription factor 4; CHOP: C/EBP homologous protein; CIRP: Cold-inducible RNA-binding protein; dpi: Day post TBI; eCIRP: Extracellular cold-inducible RNA-binding protein; ELISA: Enzyme-linked immunoassay; ER: Endoplasmic reticulum; ERS: Endoplasmic reticulum stress; FPI: Fluid percussion injury; GAPDH: Glyceraldehyde 3-phosphate dehydrogenase; GFAP: Glial fibrillary acidic protein; GRP78: Glucose regulated protein 78; H3K9ac: Histone H3 acetylation levels at the lysine 9 site; HE: Hematoxylin and eosin; Iba-1: Ionized calcium binding adapter molecule-1; DHCA: Deep hypothermic circulatory arrest; IL-1β: Interleukin-1β; KO: Neural-specific CIRP knockout; LPS: Lipopolysaccharide; mNSS: Mice neurological severity score; NC: Negative control; NeuN: Neuronal nuclei; NSE: Neuron-specific enolase; PERK: Protein kinase RNA-like ER kinase; p-PERK: Cleaved protein kinase RNA-like ER kinase; q-PCR: Real-time quantitative polymerase chain reaction; S100B: S100 calcium binding protein B; TBI: Traumatic brain injury; TLR4: Toll-like receptor 4; TNF-α: Tumor necrosis factor-α; TUNEL: TdT-mediated dUTP nick end labeling; WB: Western blot; WT: Wild-type.

## Funding

The present work was supported by grants from the National Natural Science Foundation of China (82172124, 82130062, 82241062), the National Key Research and Development Program of China (No. 2022YFA1104604) and the Key Medical Innovation Program of the Chinese People’s Liberation Army (18CXZ026).

## Authors’ contributions

YXL, MZ and YY carried out the cell and animal experiments, and drafted the manuscript. WJL, JPL and RQY participated in animal experiments, and YC and SCL collected clinical data. JW and RLY drew the illustrations. YW, ND and QHZ performed data analysis. RMY critically reviewed the manuscript. YMY conceived and supervised the study. All authors read and approved the final manuscript.

## Ethics approval and consent to participate

The animal experiments were approved by the Ethical Committee of the General Hospital of Chinese PLA, Beijing, China (No. SYXK2019–0021). The patient study was authorized by the Ethics Committee of Chinese PLA General Hospital, Beijing, China (No. S2021–539-01).

## Consent for publication

All authors approved publication of the final manuscript.

## Conflict of interest

None declared.

## Data availability

All the datasets in the manuscript or additional files are available from the corresponding authors on reasonable request.

## Supplementary Material

Supplementary_Figure_1__tkae004

Supplementary_Figure_2__tkae004

Supplementary_Figure_3__tkae004

Supplementary_Figure_4__tkae004
