# Supplementary material for: Extracellular cold-inducible RNA-binding protein mediated neuroinflammation and neuronal apoptosis after traumatic brain injury
Source: Burns Trauma. 2024 May 29;12:tkae004. doi: 10.1093/burnst/tkae004 (PMC11136617; doi:10.1093/burnst/tkae004)
Supplement: Supplementary_Figure_1__tkae004 [file supplementary_figure_1__tkae004.doc]

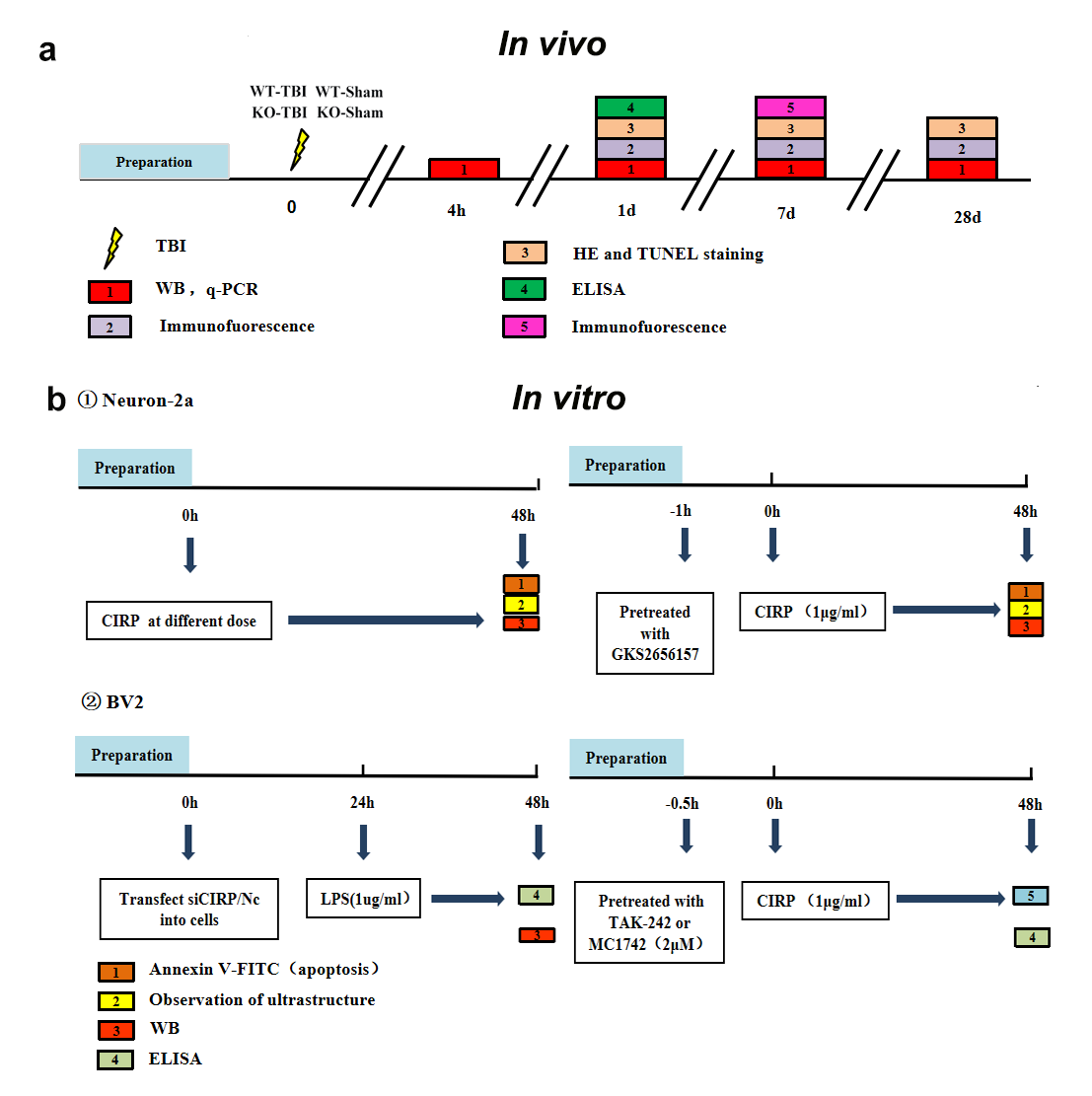
 **Supplementary Figure. 1 The chart of experimental protocols *in vivo* and *in vitro*.** (**a**). Protocol of animal experiments (TBI mice model). (**b**). Protocol of cell experiments using BV2 cells and neuron-2a cells. *CIRP* cold-inducible RNA-binding protein, *d* day, *ELISA* enzyme-linked immunoassay, *KO* neural specific CIRP knock out, *HE* hematoxylin and eosin, q-PCR real-time quantitative polymerase chain reaction *TUNE* TdT  mediated dUTP nick end labeling, *WB* Western blot, *WT* wild type
