# Supplementary material for: Extracellular cold-inducible RNA-binding protein mediated neuroinflammation and neuronal apoptosis after traumatic brain injury
Source: Burns Trauma. 2024 May 29;12:tkae004. doi: 10.1093/burnst/tkae004 (PMC11136617; doi:10.1093/burnst/tkae004)
Supplement: Supplementary_Figure_2__tkae004 [file supplementary_figure_2__tkae004.doc]

**
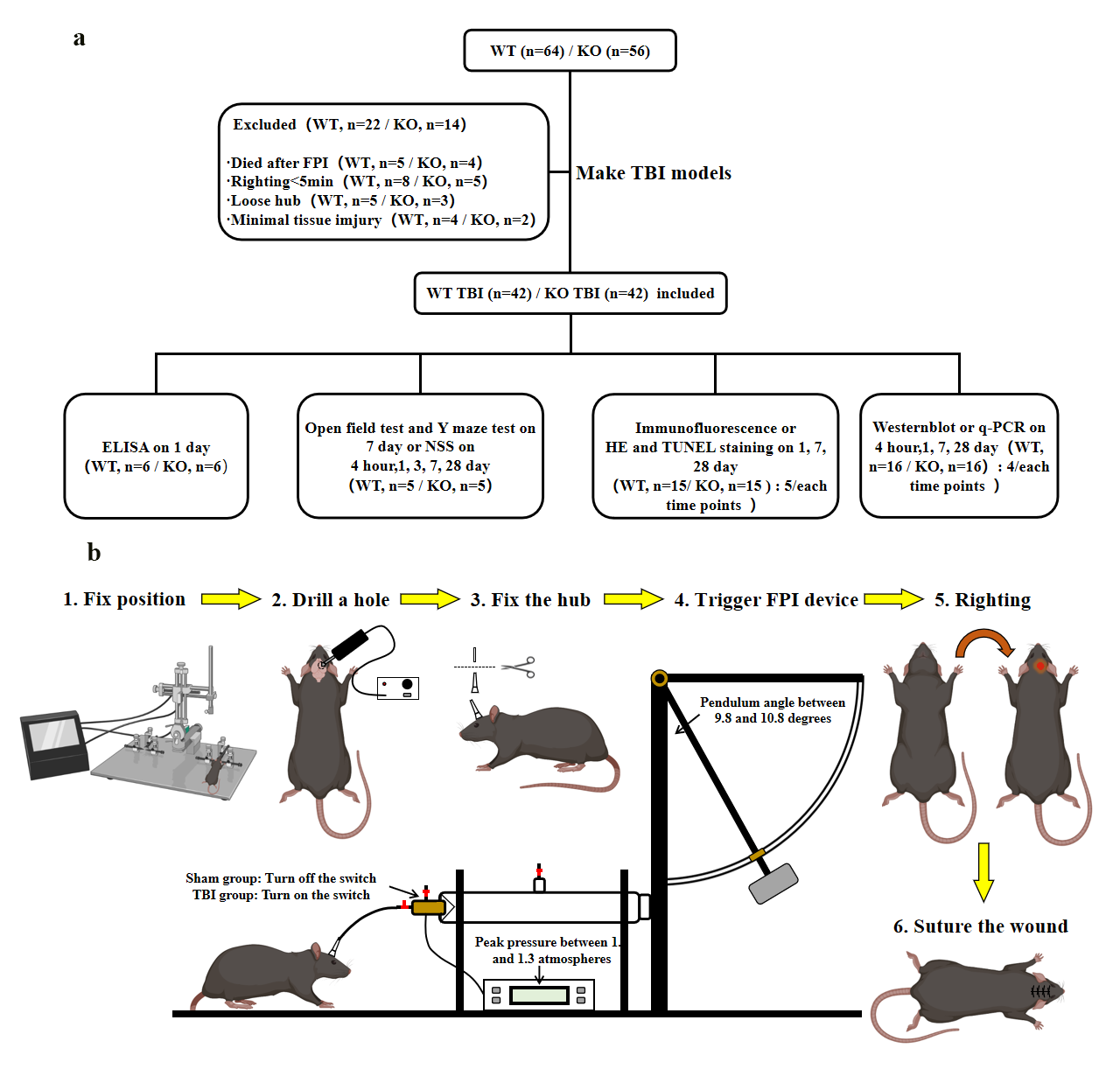
**

**Supplementary Figure. 2 The details of animal experiments.** (**a**)The design of animal experiments. The mice underwent sham or lateral FPI.Then the gene and protein expression, histological and functional changes, neurobehavioral testing,inflammatory cytokines and mediators in serum were measured at different time points after FBI. (**b**) Schematic diagram for the model of fluid percussion injury. Part of templates come from <https://www.biorender.com/>.*KO* neural specific CIRP knock out, *TBI* traumatic brain injury, *WT* wild type
