# Supplementary material for: Extracellular cold-inducible RNA-binding protein mediated neuroinflammation and neuronal apoptosis after traumatic brain injury
Source: Burns Trauma. 2024 May 29;12:tkae004. doi: 10.1093/burnst/tkae004 (PMC11136617; doi:10.1093/burnst/tkae004)
Supplement: Supplementary_Figure_3__tkae004 [file supplementary_figure_3__tkae004.doc]

**
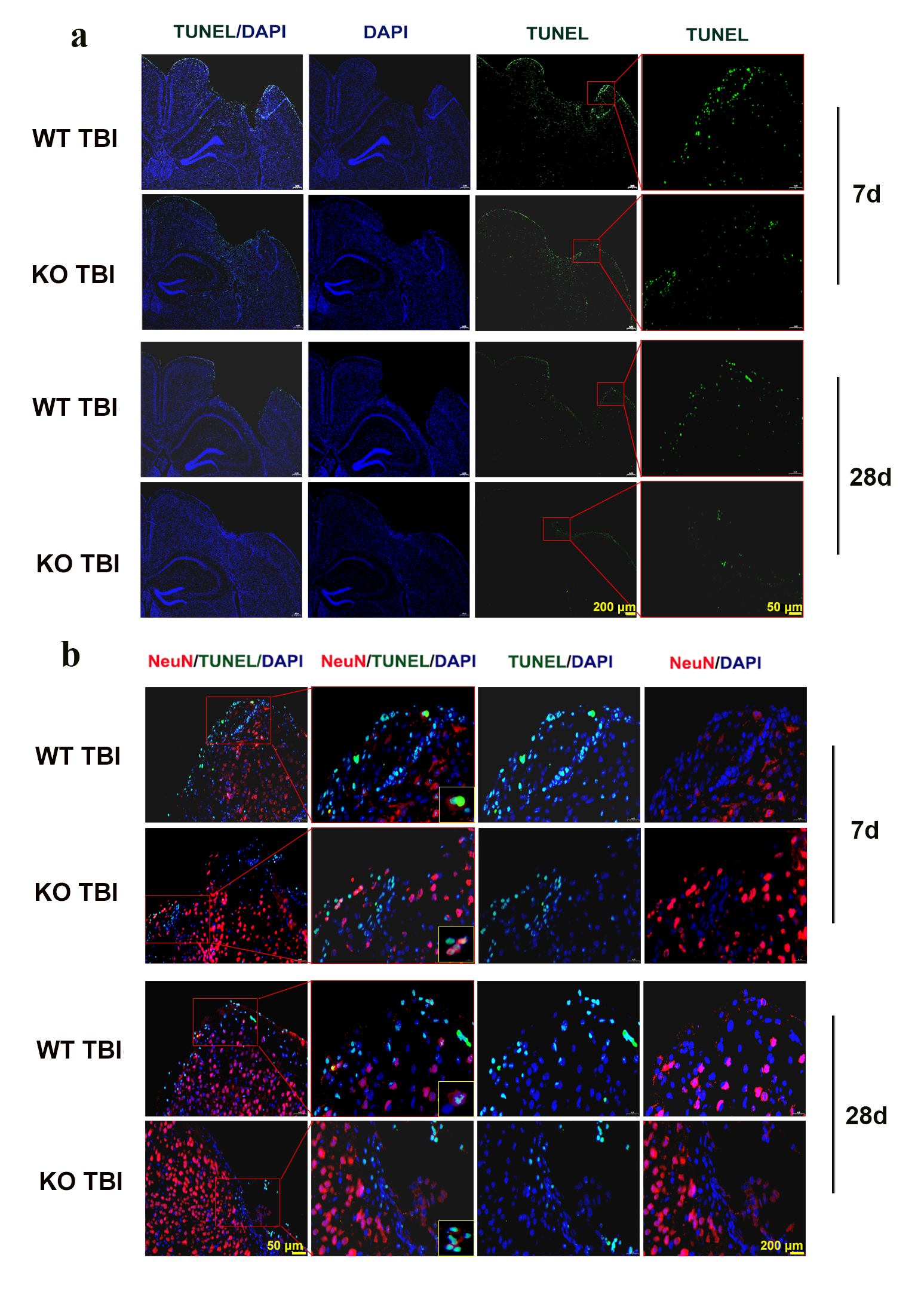
Supplementary Figure. 3 The images showed the cell apoptosis in the damage regions at different time points after TBI.** (**a**) The cell apoptosis in the damage region of the brain from WT and CIRP KO TBI mice were examined by TUNEL-stainning (green) on dpi 7,28. Scale bar=200 or 50 μm.(**b**) The neuronal cell cell apoptosis in the damage region of the brain from WT and CIRP KO TBI mice were examined by TUNEL-stainning (green) or Immunofluorescence analysis with NeuN antibody (red) on dpi 7, 28. Scale bar= 50 or 20 μm. *d* day, *DAPI* 4',6-diamidino-2-phenylindole, *KO* neural specific CIRP knock out, *NeuN* neuronal nucle *TUNEL*T dT mediated dUTP nick end labeling, *WT* wild type
