# Supplementary material for: Extracellular cold-inducible RNA-binding protein mediated neuroinflammation and neuronal apoptosis after traumatic brain injury
Source: Burns Trauma. 2024 May 29;12:tkae004. doi: 10.1093/burnst/tkae004 (PMC11136617; doi:10.1093/burnst/tkae004)
Supplement: Supplementary_Figure_4__tkae004 [file supplementary_figure_4__tkae004.doc]

**
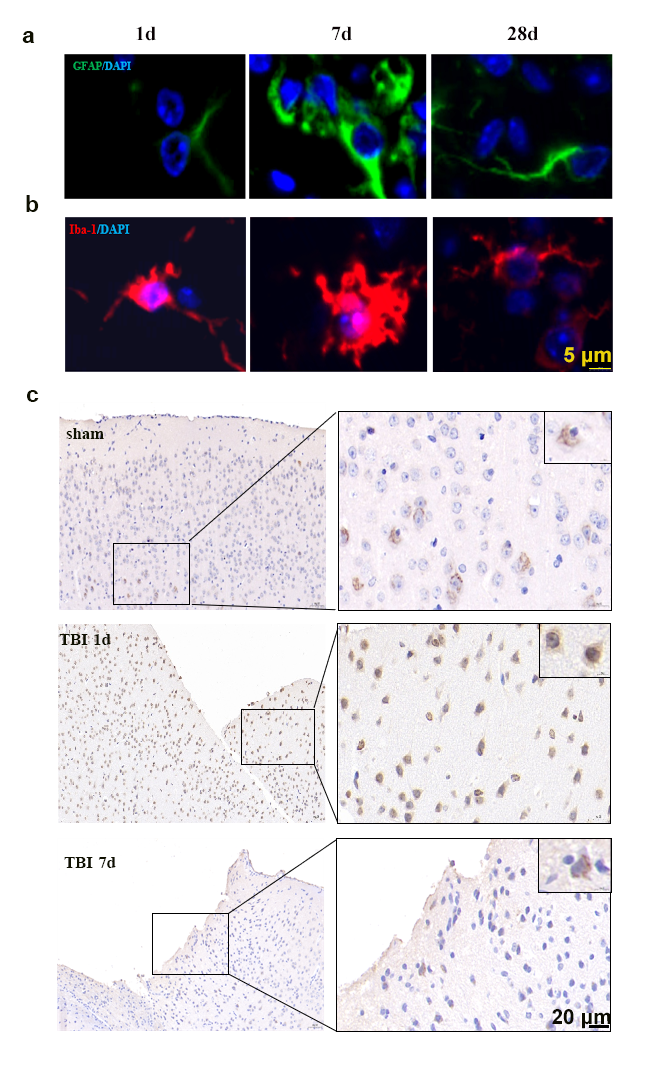
Supplementary Figure. 4 The images showed the morphology of GFAP or Iba-1 positive cells or CIRP expression in damaged cortex at different time points after TBI.** (**a**) Astrocytes or (**b**). microglia cells were detected by immunohistochemical analysis with GFAP antibody (green) or Iba-1 antibody (red) in the damaged cortex at different time points after TBI. Bar=5μm. (**c**) The location of CIRP in neural cells was detected by immunohistochemistry staining on 1 and 28 day after TBI . Bar=20 μm.*CIRP* cold-inducible RNA-binding protein, *d* day, *DAPI* 4',6-diamidino-2- phenylindole, *GFAP* glial fibrillary acidic protein, *Iba-1* ionized calcium binding adapter molecule-1,*TBI* traumatic brain injury.
